# Lessons from cross-pathogen studies: understanding the metabolic rewiring of macrophages upon infection

**DOI:** 10.3389/fcimb.2025.1584777

**Published:** 2025-06-18

**Authors:** Marisol Perez-Toledo, Alba Llibre

**Affiliations:** ^1^ Department of Immunology and Immunotherapy, School of Infection, Inflammation and Immunology, College of Medicine and Health, University of Birmingham, Birmingham, United Kingdom; ^2^ Department of Inflammation and Ageing, School of Infection, Inflammation and Immunology, College of Medicine and Health, University of Birmingham, Birmingham, United Kingdom

**Keywords:** *Mycobacterium tuberculosis*, *Salmonella* typhimurium, macrophage, metabolism rewiring, host-direct therapies

## Abstract

Bacterial infections remain a significant cause of morbidity and mortality globally. Compounding the issue is the rise of antimicrobial-resistant strains, which limit treatment options. Macrophages play key roles in the immunity and pathogenicity of intracellular infections, such as those caused by *Mycobacterium tuberculosis* and *Salmonella*. Recent advancements have enabled us to better understand how the host orchestrates immune responses to fight these infections and, specifically how the infected cell rewires its metabolism to face this challenge. The engagement of the host cell in specific metabolic pathways impacts cell function and behaviour, and ultimately, infection outcomes. In this perspective, we summarise key findings regarding the metabolic adaptations in macrophages induced by *Mycobacterium tuberculosis* and *Salmonella* infections. We also explore how cross-pathogen studies can deepen our insights into infection biology to improve therapeutic design.

## Introduction

Bacterial infections are significant contributors to deaths worldwide. The most recent report from the World Health Organization highlights that in 2019, approximately 13.7 million deaths were due to 33 bacterial pathogens, collectively making bacterial infections the second leading cause of death globally (Ikuta et al., 2022). Amongst these, infections caused by intracellular bacteria are major contributors, including *Mycobacterium tuberculosis* (*M.tb*), *Chlamydia trachomatis*, *Listeria monocytogenes* and *Salmonella enterica* (World Health Organization, 2024; Kirk et al., 2015). In this review, we focus on *M.tb* and *Salmonella enterica*, since together they are responsible for almost 20 million cases yearly, with nearly 1.5 million deaths (World Health Organization, 2023). Moreover, both microorganisms share similarities in the immune response they elicit. For example, the induction of IFN-γ^+^ CD4 T cells and the formation of granuloma-like structures are hallmark features of infection. An inability to induce these signature responses leads to uncontrolled pathogen growth in both infections (Perez-Toledo et al., 2020; Ramirez-Alejo and Santos-Argumedo, 2014), which highlights the importance of macrophages in the containment of infection.

Elie Metchnikoff discovered macrophages and their phagocytic capacity at the end of the 19th century (Metchnikoff, 1905). Since then, cumulative research has evidenced the vast macrophage heterogeneity. Different macrophage subsets in distinct body locations and at a specific moment will exist within the spectrum of fighters against infection and guardians of homeostasis. Research on how intracellular pathogens infect and survive within macrophages can reveal novel insights into the fundamental biology of such host cells. Lately, the increased interest in understanding the contribution of metabolism in macrophage physiology has also started to shed light on the role of immunometabolism in infection control. Specifically, the process of autophagy enables cell survival and proper cellular function, bridging quality control, cellular metabolism and innate immune defences. Studies in different pathological contexts have started to elucidate how autophagy and glycolysis may modulate each other. In ageing, autophagy can reset glycolysis, boosting the regenerative potential of old hematopoietic stem cells (Dellorusso et al., 2024). Our understanding of the crosstalk between autophagy and metabolic rewiring in infection settings is limited. Studies on viral immunity have reported autophagy positively (Lee et al., 2020) and negatively (Oh et al., 2021) regulating glycolysis. In this paper, we will discuss current advances in metabolism in the context of *Salmonella* and *M.tb* infection to try and complement our understanding of infection biology by comparing two microorganisms that seem so different and are yet so similar in the responses they elicit.

## M.tb rewires carbohydrate and lipid metabolism in infected macrophages

The lungs of *M.tb*-infected hosts present high glycolytic activity, evidenced by transcriptomic, proteomic and metabolomic studies quantifying expression of glycolytic enzymes and lactate, the end-product of glycolysis (reviewed in Llibre, Grudzinska et al. (Llibre et al., 2021). Studies using avirulent (HR37Ra, Bacille Calmette-Guerin) or γ-irradiated mycobacteria (e.g. iH37Rv) suggested a shift towards predominantly glycolytic macrophages upon infection (MOI 1-5) (Gleeson et al., 2016; Braverman et al., 2016). This was assessed by bioenergetic profiling (i.e. extracellular flux analysis via the Seahorse platform), and by performing lactate measurements in supernatants as a surrogate of glycolytic activity. More recent studies have shed light on the intricacies and nuances of this macrophage metabolic rewiring (Brown et al., 2025). Glycolysis is critical for effective macrophage TB immunity, and virulent *M.tb* has evolved specific prevention strategies (Cumming et al., 2018; Hackett et al., 2020; Olson et al., 2021). Mechanistically, glycolysis might be essential to produce specific antimycobacterial molecules such as IL1β or nitric oxide (NO) (Tannahill et al., 2013; Gleeson et al., 2016; Huang et al., 2018; Hackett et al., 2020) or to trigger and/or regulate specific immune defence pathways such as autophagy via lactate (O Maoldomhnaigh et al., 2021; Sun et al., 2023). This glycolytic engagement by the infected macrophage helps the host clear the infection, and the strategies employed by *M.tb* to circumvent this metabolic shift might be key for mycobacterial persistence.

**Figure 1:**
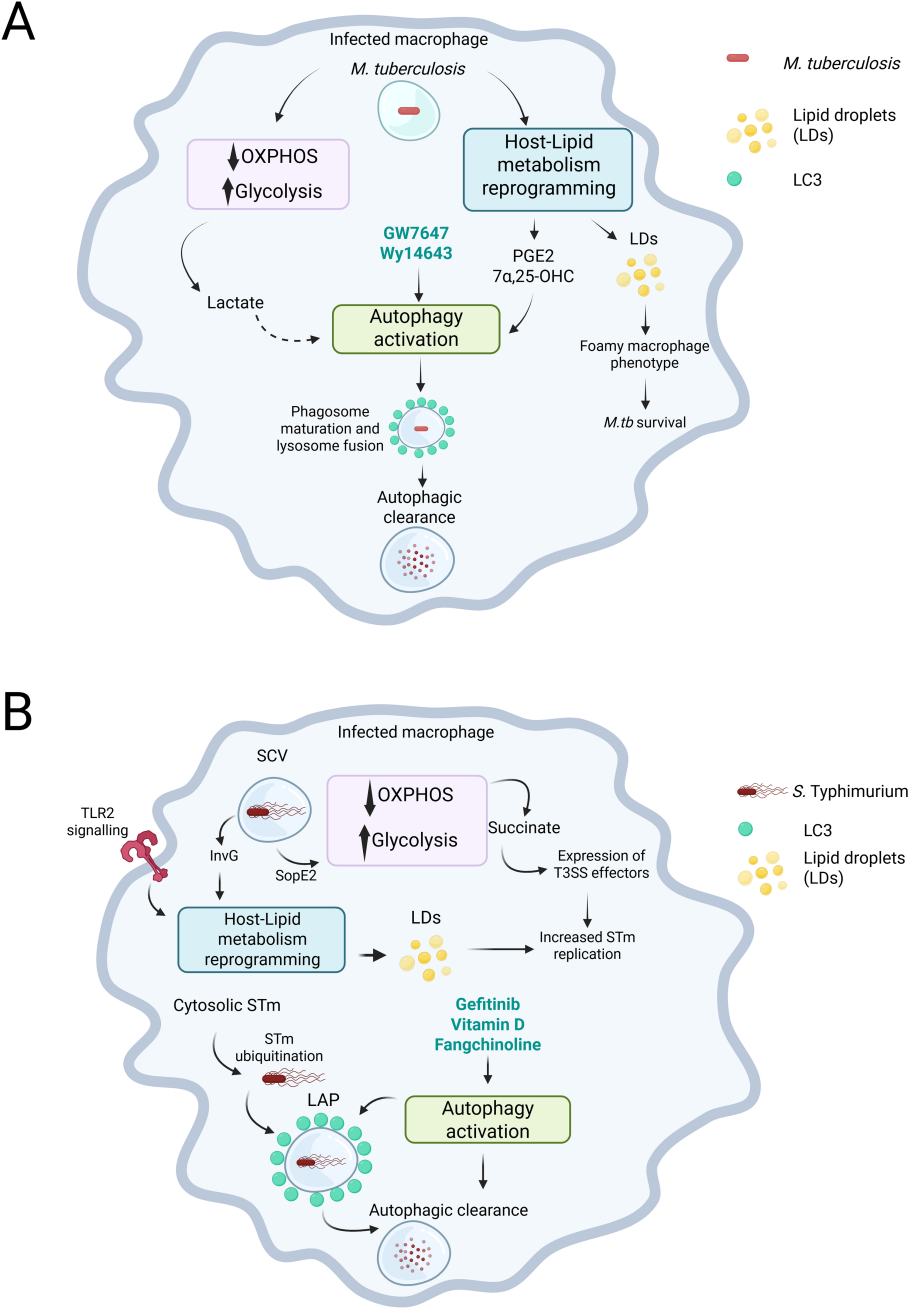
*M. tuberculosis* (*M.tb*) and *S*. Typhimurium (STm) induce metabolic changes in infected macrophages that impact infection outcomes. **(A)** In macrophages, infection with *M.tb* decreases the reliance on oxidative phosphorylation (OXPHOS) while enhancing glycolysis. This metabolic shift leads to increased levels of metabolites, such as lactate. Additionally, *M.tb* infection causes a reprogramming of lipid metabolism in the host. This creates a dichotomy: on one hand, the reprogramming results in the production of lipid droplets, which *M.tb* can exploit to survive within the infected macrophage. On the other hand, it can lead to the production of prostaglandin E2 (PGE2) or the oxysterol 7-α,25-dihydroxycholesterol (7-α,25-OHC), both of which can promote the activation of autophagy. Moreover, agonists of the PPARα receptor, such as GW7647 and Wy14643, can also stimulate autophagy. Although not directly shown in the context of *M.tb* infection, lactate has the potential to activate autophagy, which is an important process for bacterial control. **(B)** In the infected macrophage, STm is located within the *Salmonella*-containing vacuole (SCV). By expressing type III secretion system (T3SS) effectors, such as SopE2, STm alters the macrophage’s metabolism, shifting it towards glycolysis, which leads to an accumulation of succinate. STm senses the succinate and respond by promoting the expression of additional T3SS effectors to enhance its replication. Additionally, through TLR2 signalling and the action of another T3SS effector, InvG, STm can reprogram the host’s lipid metabolism to exploit it and further boosts its replication. Ultimately, compounds that promote autophagy, such as gefitinib, Vitamin D, and fangchinoline, can promote bacterial killing through the induction of LC3-associated Phagocytosis (LAP). Created in BioRender. Perez toledo, M. (2025) https://BioRender.com/y2oepra.

Lipid metabolism is profoundly reprogrammed upon *M.tb* infection, inhibiting catabolic pathways and activating *de novo* lipid synthesis and uptake [reviewed in Laval et al. (Laval et al., 2021)]. This causes the development of a foamy, lipid-rich macrophage phenotype, which is a hallmark of TB granulomas (D’Avila et al., 2006; Peyron et al., 2008). There is strong evidence supporting the use of host lipids (cholesterol and fatty acids) by *M.tb* to eat (Pandey and Sassetti, 2008), survive (Daniel et al., 2011) and specifically impair key host immune defence mechanisms (e.g. autophagy, phagosome maturation) (Singh et al., 2012; Chandra and Kumar, 2016) (**Figure 1**). Therefore, on one hand, the mycobacterial-induced host lipid rewiring enables pathogen survival and immune evasion. On the other, lipid droplets and lipid mediators (e.g. prostaglandin E2, Leukotriene B4) are key in generating effective anti-mycobacterial responses within the macrophage (Mayer-Barber et al., 2014; Mayer-Barber and Sher, 2015; Bosch et al., 2020; Sorgi et al., 2020). Furthermore, cholesterol-derived compounds such as oxysterols and vitamin D can restrict mycobacterial growth (Bah et al., 2017; Varaksa et al., 2021; Foo et al., 2022). Thus, the host lipid metabolic adaptation in response to infection is also essential for effective protective responses. The specific factors that tilt the balance in favour of the host or the pathogen are still not fully understood. Other relevant metabolic adaptations (e.g. amino acids, TCA cycle intermediates, ions) are beyond the scope of this *Perspective* and have been excellently reviewed elsewhere (Neyrolles et al., 2015; Hackett and Sheedy, 2020).

The new macrophage metabolic landscape triggered by infection results in changes regarding the accumulation and availability of specific metabolites. These are the primary sources of post-translational modifications and epigenetic regulation, which regulate essential cellular processes such as autophagy (Sun et al., 2023; Huang et al., 2024; Shu et al., 2023; Nieto-Torres et al., 2023; Park et al., 2022). Thus, the predominance of specific metabolic pathways will dictate gene expression profiles that may impact cell function and behaviour and, ultimately, infection outcome.

## Salmonella rewires carbohydrate and lipid metabolism in infected macrophages

*Salmonella enterica* are important pathogens for human and animal health. Notably, serovars Typhimurium (STm) and Enteritidis are linked to non-typhoidal salmonellosis (NTS), which occasionally develops into invasive disease (iNTS), especially in young children and immunocompromised individuals, with mortality rates reaching up to 25% (Feasey et al., 2012). As with *M. tb*, the interaction between STm and macrophages has a profound impact on the successful establishment of infection and the outcome for the host.

Recent research has highlighted how STm affects metabolic reprogramming and the subsequent impacts on infection outcomes. *In vitro*, through the *Salmonella* Pathogenicity Island-I (SPI-I) effector SopE2, STm represses serine synthesis in peritoneal macrophages. The result is an increase in glycolysis, which is accompanied by a significant accumulation of glycolytic products, such as pyruvate, lactate, and phosphoglycerates, as well as TCA intermediates, such as fumarate, α-ketoglutarate, and succinate (Jiang et al., 2021). The bacteria can then uptake some of these intermediates and use them as carbon sources, which results in increased intracellular replication. Moreover, this effect has also been shown *in vivo*, where mice infected with STm and supplemented with lactate showed increased bacterial burdens in the spleen and liver 3 days post-infection (Wang et al., 2023). The authors attributed this effect to a switch of macrophages towards an M2-like anti-inflammatory phenotype, which has been associated with more permissiveness to STm infection (Pham et al., 2020; Panagi et al., 2020). Recent work by Rosenberg et al. reported that succinate, which accumulates due to the macrophage glycolytic shift and the concomitant truncation of the TCA cycle, is sensed by STm and induces the expression of type 3 secretion system (T3SS) effectors, such as *sseL*, *steC*, *ssrB*, and *pipB2*, resulting in increased bacterial burdens (Rosenberg et al., 2021). However, not everything is bad news for the host. Khatib-Massalha et al. showed that infection with STm increases lactate levels in the bone marrow. This increase leads to increased permeability of the bone marrow, thus increasing neutrophil mobilisation through lactate signalling via the GPR81 receptor (Khatib-Massalha et al., 2020).

Similarly to what has been observed with *M.tb* infection in macrophages, STm can also interact with the mammalian lipid metabolism to induce the production of lipid droplets (LDs). Experiments with monocyte-derived human macrophages supplemented with oleic acid showed that STm infection induced the production of LDs shortly after infection. However, this did not result in better control of bacterial infection (Bosch et al., 2020). A more recent study elegantly dissected the mechanism of LDs formation following STm infection, which required the T3SS effector *invG* and TLR2 signalling (Kiarely Souza et al., 2022). Moreover, this work also showed that the bacteria may benefit from forming LDs, as pharmacologically inhibiting key enzymes involved in forming LDs reduced bacterial burdens (**Figure 1**) (Kiarely Souza et al., 2022).

So far, most of the evidence shown here suggests a deleterious role for macrophages engaging in glycolysis in the outcome of STm infection, contrasting with the existing evidence in *M.tb* infection. However, this could be the result of an oversimplification of the models used, e.g. Most studies shown here use human or mouse-derived bone marrow macrophages. As not all macrophages are the same (Huang et al., 2018; Gordon and Taylor, 2005; Heieis et al., 2023; Russell et al., 2025), future studies could benefit from considering how the intrinsic differences in macrophage populations can affect their response to metabolic changes induced by pathogens. However, both STm and *M.tb* appear to exploit the changes in lipid metabolism within infected macrophages to their advantage. Further research on this shared pathway will be crucial for identifying potential targets for infection control.

## Autophagy in the context of intracellular pathogens such as STm and M.tb

In the previous sections, we have summarised some key immune-metabolic events in host macrophages after *Salmonella* and *M.tb* infection, including changes in glycolysis and lipid metabolism. Immune metabolism comprises the metabolic adaptations of the host cell to face challenges such as infection. Additionally, it covers how cellular engagement on particular metabolic pathways results in the accumulation of specific metabolites (e.g. lactate, succinate, prostaglandins, oxysterols), which can directly act as signalling molecules, fuel, and/or substrates for post-translational and epigenetic modifications. Furthermore, metabolism and immunity converge in essential biological processes to regulate and tackle cellular stress. The process of autophagy perfectly illustrates this. Also known as self-eating, this cellular housekeeping strategy clears the cytoplasm from waste and defective macromolecules and organelles, acting as a quality control for the cell and enabling survival. Autophagy is not only key for metabolic homeostasis but also constitutes an innate immune defence mechanism of paramount relevance for controlling intracellular pathogens, directing them to autophagolysosomal degradation.

The role of autophagy as a key mechanism of *M.tb* defence within macrophages was first described using a combination of RAW cells (Herb et al., 2024) and murine and human monocyte-derived macrophages (Gutierrez et al., 2004). Since then, different studies have assessed the interplay and function of canonical and non-canonical autophagy in the immune responses of macrophages to *M.tb* infection. Evidence for both protective (Golovkine et al., 2023) and non-protective (Kimmey et al., 2015) roles of autophagy in the outcome of *M.tb* infection has been described. Similarly, autophagy has been reported to be an essential mechanism for intracellular control of STm. STm is targeted by autophagy when bacteria escape the *Salmonella*-containing vacuole (SCV) into the cytosol. This triggers the ubiquitination of cytosolic bacteria and the recruitment of LC3 and other autophagy proteins, restricting intracellular bacterial growth (Birmingham et al., 2006). However, STm can also exploit the autophagy machinery to promote SCV maturation, thus inducing the activation of T3SS effectors that supports bacterial replication (**Figure 1**) (Kreibich et al., 2015).

The macrophage metabolic rewiring that occurs upon *M.tb* and *Stm* infection has the potential to modulate autophagy. For instance, the formation and maturation of autophagosomes, as well as endosome-lysosomal degradation need lactylation of specific core autophagy proteins (e.g. PIK3C3/VPS34, TFEB, a master regulator of autophagy) (Sun et al., 2023; Huang et al., 2024). Although not specifically assessed in the context of infection, lactate has been shown to promote autophagy in different contexts, including retinal degradation (via upregulation of the LCII/II ratio), high intensity interval training (via ERK1/2/mTOR/p70S6K activation, and neurodegeneration (through cytosol acidification) (Zou et al., 2023; Pirani et al., 2024; Fedotova et al., 2022). Distinct lipid mediators also have the potential to modulate autophagy. For example, LTB4 suppressed autophagy in mouse lung macrophages (Zhang et al., 2019a), and PGE2 induced autophagy in PBMCs from healthy donors and TB patients stimulated with *M.tb* cell lysate (Pellegrini et al., 2021). The oxysterol 7α,25-dihydroxycholesterol (7α,25-OHC) reduced mycobacterial growth through increased autophagy via GPR183 signalling in human monocytes (Bartlett et al., 2020). These examples illustrate how infection triggers host cell’s metabolic adaptations, resulting in changes in the intracellular pool of available metabolites which can regulate key biological/immunological processes such as autophagy.

Researchers from both the TB and *Salmonella* fields have exploited the zebrafish model to elucidate both mechanisms of bacterial virulence and host protective responses. The autophagy receptors p62 and Optineurin were shown to be essential for protection against mycobacterial infection and to act independently (Zhang et al., 2019b; Xie and Meijer, 2023). However, the same p62 and Optineurin receptors were shown to be interdependent when promoting autophagy in the context of *Salmonella* infection. Lc3-associated phagocytosis (LAP) was identified as the primary autophagy-related process behind macrophage control for STm infection (Masud et al., 2019a, 2019b). Although the specific role of LAP in the context of mycobacterial infection has not been tested explicitly in the zebrafish model, LAP could not clear *M.tb* infection in a mouse model of TB (Koster et al., 2017). We can learn much about the biological nuances of crucial immune defence mechanisms (i.e., autophagy) by using the same model but different pathogens or distinct models with the same pathogen. Although further research is needed, distinct roles for specific components of the autophagy machinery might be pathogen-specific, knowledge that could be harnessed therapeutically.

## Discussion

The threatening increase in antimicrobial resistance has prompted the search for alternative therapeutic strategies, including host-directed therapies (HDTs). In contrast to conventional antibiotic drugs, which target the pathogen, HDTs modulate specific host factors to pursue various aims. For instance, we might want to dampen inflammation to prevent immune-driven tissue pathology, enhance the bactericidal capacity of the infected macrophage, or disrupt the granuloma structure to expose the bacteria to conventional antibiotic treatments (Young et al., 2020). HDTs have dramatically changed the treatment and outcomes of specific cancers (i.e. checkpoint inhibitors such as anti-PD1 or anti-CTLA4 antibodies) (Robert, 2020); unfortunately, their use in infectious diseases has limitedly reached clinical practice.

The first step in designing and using novel HDTs is to have an accurate mechanistic understanding of the host pathway that needs targeting. For instance, anti-mycobacterial autophagy can be promoted via modulation of different host factors, including nuclear receptors such as peroxisome proliferator-activated receptor (PPAR) and estrogen-related receptor (ERR) (Kim et al., 2019), vitamin D (Yuk et al., 2009; Campbell and Spector, 2012) and AMPK (Yang et al., 2014; Singhal et al., 2014; Parihar et al., 2014; Guerra-De-Blas et al., 2019). Specifically, the compounds GW7647 and Wy14643, which are PPARα activators, have been shown to successfully reduce bacterial burdens in *M. tb*-challenged bone marrow derived macrophages, presumably through the activation of autophagy and increased phagosome maturation (Kim et al., 2017). Although these specific PPAR agonists have not been evaluated in the context of STm infection, there is evidence that suggests that PPAR inhibition instead of activation results in better bacterial control and reduced immunopathology (Taddeo et al., 2024). However, the use of an inverse agonist of ERRγ (GSK5182) resulted in lower intracellular burdens in STm infected macrophages, although this was the result of iron metabolism modulation, which is out of the scope of our paper (Kim et al., 2014). Vitamin D induced intestinal epithelial autophagy during *Salmonella* infection (Huang, 2016; Huang and Huang, 2021). The Epidermal growth factor receptor (EGFR) inhibitor Gefitinib reduced *Salmonella* survival in macrophages and mice via host metabolic reprogramming, including autophagy (Sadhu et al., 2021). Also, fangchinoline, a phytochemical with anti-proliferative properties, promoted *Salmonella* killing *in vitro* and *in vivo* through autophagy (He et al., 2025). While most studies were performed *in vitro* using a combination of monocytic cell lines and primary mouse or human macrophages, some of them used *in vivo* models, including mice and zebrafish (reviewed in Adikesavalu et al. and Wu et al. (Wu et al., 2020; Adikesavalu et al., 2021). Furthermore, molecules such as vitamin D and AMPK modulators, such as metformin, have been and continue to be tested in clinical trials. Some (not all) studies show promising results in using these compounds in combination with conventional antibiotic treatment to improve TB outcomes (Salahuddin et al., 2013; Padmapriydarsini et al., 2022; Sutter et al., 2022; Biswal, 2021). Still, more research is needed to find effective HDTs to aid the fight against TB.

The terminology we use in science significantly shapes our understanding of the entities we study. Terms like T cells, B cells, and macrophages can limit our perspective, especially when new cell types are discovered that do not fit into existing classifications. This issue also affects how we approach the study of diseases. In cancer, for instance, concentrating on tumour types based on their tissue location may be less beneficial than focusing on the underlying mechanisms that cause the cancer, such as mutations in the p53 or BRCA genes. Consequently, therapies aimed at addressing the pathological mechanisms driven by mutations in p53 could potentially be applied to various cancer types associated with this same mutation. A comprehensive, mechanistic understanding of the metabolic rewiring undergone by host macrophages upon infection, and how these shape protective responses, is a first key step for the identification of novel host therapeutic targets. Furthermore, the way we use language regarding pathogens and the host cellular pathways they elicit, could also open new avenues for novel treatments. For instance, instead of referring to infections caused by *Mycobacteria*, *Salmonella*, *Burkholderia*, or *Leishmania*, we could categorise them, as pathogens that promote lipid droplet formation to their advantage, (e.g. STm and *M. tb*), or as pathogens that are LAP-susceptible or LAP-resistant. This shift in how we use language, driven by a deeper understanding of the fundamental biological changes triggered by infection, could revolutionize the development or repurposing of host-directed therapies for infections that continue to claim millions of lives worldwide each year.

## Data Availability

The original contributions presented in the study are included in the article/supplementary material. Further inquiries can be directed to the corresponding authors.
